# Primary Congenital Lymphedema Complicated by Hydrops Fetalis: A Case Report and Review of the Literature

**DOI:** 10.1155/2013/186173

**Published:** 2013-02-28

**Authors:** Paul Singh, Matthew Connell

**Affiliations:** ^1^Division of Maternal-Fetal Medicine, Department of Obstetrics and Gynecology, University of Missouri School of Medicine, 2301 Holmes Street, Kansas City, MO 64064, USA; ^2^Department of Obstetrics and Gynecology, University of Missouri School of Medicine, 2301 Holmes Street, Kansas City, MO 64064, USA

## Abstract

*Introduction*. Primary congenital lymphedema is a rare disorder associated with insufficient development of lymphatic vessels. Usually most patients present with lower extremity edema seen sonographically. Rarely primary congenital lymphedema may be associated with severe lymphatic dysfunction resulting in hydrops fetalis. *Case*. A 27-year-old primigravida with a family history of leg swelling throughout multiple generations was diagnosed early in the third trimester with hydrops fetalis. Delivery was undertaken at 32 weeks for nonreassuring fetal status and the infant expired at approximately 45 minutes of life. Primary congenital lymphedema was confirmed via molecular testing of the vascular endothelial growth factor receptor-3 gene. *Discussion*. The diagnosis of PCL is suspected prenatally when ultrasound findings coincide with a positive family history of chronic lower limb lymphedema. Isolated PCL is rarely associated with significant complications. Rarely, however, widespread lymphatic dysplasia may occur, possibly resulting in nonimmune hydrops fetalis.

## 1. Introduction

Primary congenital lymphedema (PCL), also known as Milroy syndrome, is named after William Forsyth Milroy who described a congenital chronic state of lymphedema of the lower extremities in six generations of an affected family [1]. Although often referred to Milroy syndrome, cases of congenital hereditary elephantiasis were also described by Nonne one year earlier in 1891 [2]. PCL has an estimated incidence of 1 in 33,000 live born infants and typically affects more females than males. Generally PCL presents prenatally with lower extremity edema seen on ultrasonography; however, more extensive lymphedema involving the upper limbs, thorax, and facial regions have been reported [3–6]. We describe a unique case of confirmed PCL complicated by hydrops fetalis.

## 2. Case Presentation

**Figure 1:**
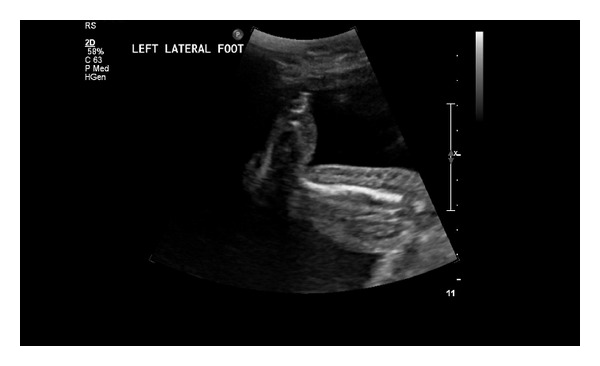
Note the swelling present along the dorsal aspect the left foot and throughout the distal left lower extremity.

**Figure 2:**
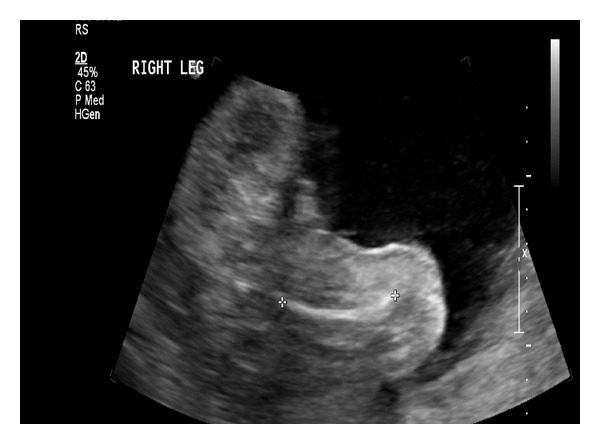
Note the swelling along the distal right lower extremity.

**Figure 3:**
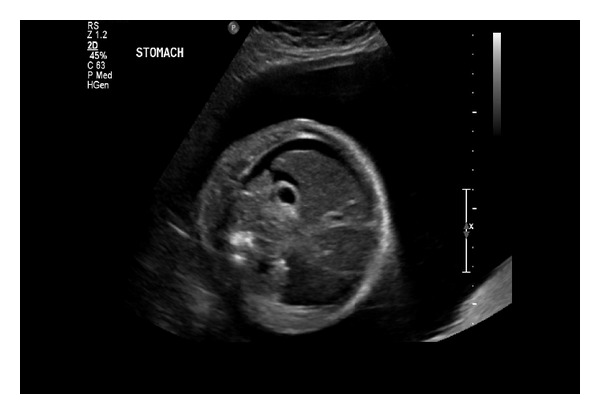
Cross sectional view of the abdomen showing abdominal ascites.

**Figure 4:**
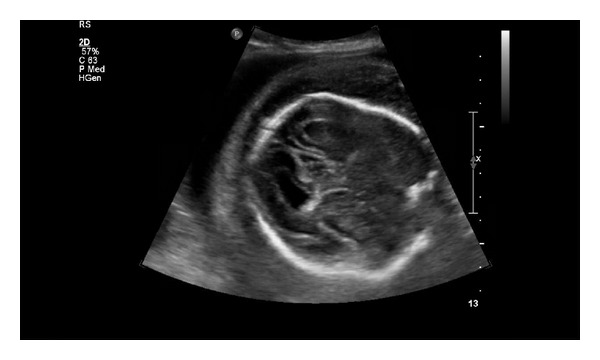
Cross sectional view of the fetal head showing skin edema.

A 27-year-old primigravida underwent a screening ultrasound at 28 and 5/7 weeks gestation. The scan revealed edema of the lower extremities that was particularly evident on the calves and feet (Figures 1 and 2). Other sonographic findings included abdominal ascites (Figure 3) and scalp edema (Figure 4). The patient's medical history was unremarkable aside from a family history of “leg swelling” throughout multiple generations. A workup for hydrops was performed, including a complete blood count, indirect Coombs testing, fetal echocardiography, hemoglobin electrophoresis, and serologic testing for congenital infections, all of which were normal. An amniocentesis was performed that revealed a normal female karyotype. Antepartum testing at 32 weeks gestation demonstrated a biophysical profile score of 4/10 and Cesarean delivery was performed. The infant was born with extreme anasarca without spontaneous respirations. Despite intubation and aggressive resuscitation, the neonate expired after approximately 45 minutes of life. The diagnosis of PCL was proposed on the basis of family history and clinical findings, and was confirmed by molecular analysis showing the c.3109 G > C mutation in the vascular endothelial growth factor receptor-3 (VEGFR3) gene. 

## 3. Discussion

Hereditary lymphedema is an inherited disorder resulting in chronic tissue swelling caused by abnormal lymphatic drainage. There are three subtypes of hereditary lymphedema: primary congenital lymphedema (PCL) which presents at birth, lymphedema praecox which manifests during childhood, and lymphedema tarda that occurs as an adult [7]. PCL exhibits an autosomal dominant inheritance pattern with a reported penetrance of 80–84% [8, 9]. The extent and severity of the edema, however, are highly variable and can vary significantly even within the same family. 

Multiple gene loci may be involved in the causation of PCL [10]. In particular, the vascular endothelial growth factor receptor-3 (VEGFR3) gene has been implicated in the pathogenesis of PCL. Located on the long arm of chromosome 5 (5q34-5q35), VEGF receptor-3, also known as FLT4, is expressed in the endothelium of lymphatic tissues. Normally, vascular endothelial growth factors VEGF-C and VEGF-D bind to VEGFR-3 in order to promote lymphatic vasculogenesis and angiogenesis [11–14]. Mutations interfering with the VEGFR-3 tyrosine kinase signaling function are thought to result in PCL [15]. Lymphatic flow, thus, becomes interrupted, leading to intravascular and interstitial accumulation of lymph and tissue fluid. PCL may be associated with a variety of syndromes, including Noonan, Turner, yellow-nail, and lymphedema-distichiasis syndrome.

The diagnosis of PCL is suspected prenatally when ultrasound findings coincide with a positive family history of chronic lower limb lymphedema. The most common ultrasound finding seen in PCL is edema of the dorsal aspect of the feet [16–18]. Less common ultrasound findings that have been reported include hydrocele, dilated umbilical cord, dilated bowel loops, syndactyly, ambiguous genitalia, increased nuchal translucency, and hydrothorax [17, 19, 20]. Prenatal findings suggestive of PCL warrant a targeted ultrasound, fetal karyotyping, and serial limited ultrasounds to look for progression of lymphatic dysfunction as evidenced by worsening lymphedema. Spontaneous resolution of PCL associated lymphedema during fetal life has also been reported to occur [21]. Prenatal genetic testing for PCL is not widely available; however, Sanger sequencing as well as deletion/duplication testing for the VEGFR3/FLT4 gene is available. Delivery may be carried out at term with Cesarean section reserved for obstetrical indications only. 

Isolated PCL is rarely associated with significant complications postnatally and delivery need not to be carried out in a high acuity perinatal center. PCL associated lymphedema is generally painless, nonpitting, and does not ulcerate or form varicosities. The edema may, however, cause disfigurement, cellulitis and papillomatosis. Treatment consists of a combination of compression, manual massage, and antibiotics. Rarely PCL may be associated with significant complications. Wheeler et al. reported a case of intestinal lymphangiectasia in a case of Familial lymphedema praecox while Offori and Brostrom et al. described cases of PCL associated with angiosarcoma and lymphangiosarcoma, respectively [22–24]. 

Our case is unique in that PCL was complicated by hydrops fetalis. Hydrops fetalis is defined as abnormal accumulation of fluid in two or more fetal compartments, including ascites, pleural effusion, pericardial effusion, skin edema, polyhydramnios, and placental edema. The mechanism for the formation of fetal hydrops is an imbalance of interstitial fluid production and its subsequent lymphatic return. Prior to the introduction of immunoglobulin prophylaxis for at-risk mothers, immune-mediated hydrops predominated. Today, however, nonimmune hydrops accounts for the majority of reported cases. Nonimmune hydrops fetalis (NIH) is a heterogeneous disorder with a variety of possible causes, including fetal arrhythmias, congenital infections, structural heart disease, alpha thalassemia, aneuploidy, sacrococcygeal teratomas, and twin to twin transfusion syndrome. Etiologies of NIH that are most commonly amenable to treatment include fetal tachyarrhythmias and congenital parvovirus infection and thus have a better prognosis than other causes of NIH. Generally, however, the prognosis is poor and is associated with significant perinatal morbidity and mortality [25–28]. In a retrospective study reviewing all pregnancies complicated by NIH over 10 years, Santo et al. reported a survival rate of only 48% [29]. Given the negative workup for NIH, we propose that in our case the fetus was so severely affected by PCL, that widespread lymphatic dysplasia resulted, leading to diminished lymphatic return and the development of hydrops fetalis. To our knowledge only one such case has been reported previously in the literature [21]. Indeed, Daniel-Spiegel et al. described a case of PCL complicated by massive hydrothorax [21]. In utero thoracentesis was performed prior to delivery and followup at 12 months postpartum revealed bilateral foot edema with otherwise normal development.

Although the clinical course of lymphatic dysfunction is most commonly confined to the lower extremities in PCL, awareness of the possibility of the development of nonimmune hydrops is important for clinicians and underscores the importance of serial sonography to monitor fetuses suspected to have primary congenital lymphedema. Although, in our case, the infant expired at birth, increased antepartum surveillance in patients with suspected PCL found to have NIH may improve perinatal outcomes, aid in patient counseling, and assist with delivery planning.
